# An integrated surveillance and management model for *Baylisascaris procyonis* in invasive raccoons (*Procyon lotor*) in Italy

**DOI:** 10.3389/fvets.2026.1846789

**Published:** 2026-06-17

**Authors:** Alessia Mariacher, Ziad Mezher, Paola Pepe, Andrea Caprioli, Claudio De Liberato, Claudia Eleni, Gianluca Fichi, Antonino Barone, Giuseppina Brocherel, Rita Quondam Giandomenico, Sara Greco, Laura Salvato, Rita Lorenzini, Roberta Gori, Beatrice Falorni, Nadia Cappai, Luca Mattioli, Alessio Capecci, Martina Benedetti, Andrea Lombardo

**Affiliations:** 1 Istituto Zooprofilattico Sperimentale del Lazio e della Toscana “M. Aleandri”, Rome, Italy; 2 Parco Nazionale Foreste Casentinesi, Monte Falterona e Campigna, Arezzo, Italy; 3 Regione Toscana, Firenze, Italy

**Keywords:** baylisascariosis, invasive species management, *larva migrans*, One Health, paratenic host, wildlife disease control, zoonosis

## Abstract

The raccoon (*Procyon lotor*), an invasive alien species in Europe, is a known reservoir for zoonotic parasites, particularly *Baylisascaris procyonis*, which can cause severe *larva migrans* syndromes in humans and animals. Following the first report of *B. procyonis* in the raccoon population of the Province of Arezzo, Central Italy, in 2021, we developed and implemented a One Health integrated surveillance and management approach combining carcass collection, parasitological and molecular diagnostics, GIS-based risk mapping, environmental fecal monitoring, and targeted public health outreach. During the study period (2021–2024), *B. procyonis* was detected in 34 (33.3%) of the 102 examined raccoons. No evidence of infection was identified in 169 examined paratenic hosts, while 30 wolf carcasses and 15 fecal samples from at-risk domestic dogs also tested negative, suggesting no evidence of parasite circulation outside the raccoon reservoir, within the limits of the investigated samples. Genetic characterization of raccoons revealed only one mitochondrial haplotype in all *B. procyonis* positive raccoons, suggesting a single introduction event of individuals that escaped from captivity in the early 2000s. GIS analysis identified a high-risk core area of 265 km^2^, characterized by low human activity and the absence of latrines, tracks and other signs confirming the presence of raccoons. These results highlight the importance of raccoons as reservoirs of zoonotic parasites and demonstrate the relevance of a coordinated multidisciplinary surveillance model for assessing and managing emerging wildlife-borne hazards. This integrated approach provides a blueprint for early detection and risk mitigation as raccoon populations are likely to continue expanding across Europe.

## 1 Introduction

The raccoon (*Procyon lotor*), an omnivorous species native to North America, has been introduced into several European and Asian countries, where it is now considered an invasive alien species due to its substantial ecological impact (1). Its broad dietary plasticity poses threats to biodiversity, agriculture and animal farming, including damage to crops and poultry (1). Raccoons frequently inhabit human-modified and peri-urban environments, thus increasing the potential for zoonotic pathogen transmission (2).

In Italy, first records of the raccoon in the wild date back to 2004 in Lombardy, Northern Italy, where a local introduction event led to the establishment of a reproductive population along the Adda river; this population was eradicated in 2019 (3, 4). Other several reports of raccoons have been reported, scattered across the peninsula, but most of these have involved single individuals, likely escapees from captivity, and these have not led to the formation of breeding populations (5).

A self-sustaining raccoon population has settled in the Casentino area (Arezzo Province, Tuscany, Central Italy) since 2013, originating from individuals that escaped from captivity (6). The population has expanded into the Foreste Casentinesi, Monte Falterona and Campigna National Park (43.8506° N, 11.7783° E), reaching up to the Emilia-Romagna border, with an estimated size of at least 200 individuals (Mattioli, pers. comm.). In response, an eradication program was initiated in 2016 under Regulation (EU) 1143/2014, and culled animals have since been systematically submitted for health assessment (7).

During necropsy, *Baylisascaris procyonis*, a zoonotic nematode native to North America, was first identified in nine raccoons between April and September 2021, representing the first detection of this parasite in Italy (8). Raccoons serve as the primary definitive host, while numerous paratenic hosts, including wild rodents, mustelids, lagomorphs and birds, may acquire the infection through ingestion of feces contaminated with parasite eggs (9). Dogs and other canids as wolves can occasionally act as definitive hosts (1). Because raccoons preferentially defecate in specific sites, known as *“latrines,”* these locations constitute critical sources of environmental contamination for both animals and humans (10). Infected raccoons may shed up to 20,000 eggs per gram of feces (11): the eggs undergo the maturation process in the ground, reaching the infective stage containing the typical L_3_ larvae in about 20 days (1). Human cases, although rare, have occurred in areas where latrines are located near children’s play sites (1). Human infection can lead to severe or fatal disease due to larval migration through critical tissues, including the central nervous system (12). Environmental contamination from raccoon faeces is concerning due to the long-term persistence of *B. procyonis* eggs, and can sustain the parasite’s life cycle even after host eradication (13).

Given these One Health implications, a multidisciplinary surveillance program was launched in the Arezzo Province. The project, conducted between 2021 and 2024, aimed to assess *B. procyonis* prevalence in raccoons, investigate potential involvement of local wildlife, evaluate environmental contamination, perform genetic analyses of raccoon hosts, implement human surveillance and public awareness initiatives, and develop management and decontamination guidelines. The present study describes the methodologies employed and summarizes the main outcomes of this integrated surveillance/management approach.

## 2 Materials and methods

### 2.1 Study area

The study was conducted in the Casentino Valley (approximately 43°44′N, 11°46′E), a mountainous region covering ~800 km^2^ in the province of Arezzo, Tuscany, Central Italy. The valley, which extends along the Arno river basin for 30–60 km, is characterized by heterogeneous topography, including forests, mountains, and high-yield crops such as vineyards, fruit trees, and conifer plantations with white spruce and black pine. Several small towns are distributed along the river and its tributaries, and the area includes the “Foreste Casentinesi, Monte Falterona e Campigna” National Park, a protected area of ecological importance (14).

### 2.2 Animals and sample collection strategy

A coordinated network among local wildlife management and environmental authorities was established to ensure comprehensive and harmonized sample collection. Collaborators included the Tuscany Region, provincial and forestry police units, local hunting districts, wildlife rescue centers, and the National Park Authority. Field personnel were trained in the safe handling, collection, and transport of both animal and environmental samples according to biosafety protocols.

Throughout the study period (2021–2024), the network facilitated the systematic collection of raccoon carcasses from the official eradication program or found dead from other causes. Additional samples included carcasses of wildlife species considered susceptible to *B. procyonis*, both as definitive hosts [e.g., wolves (*Canis lupus*)] and as paratenic hosts [e.g., mustelids, red foxes (*Vulpes vulpes*), lagomorphs, rodents, and wild birds]; fecal samples from privately owned dogs potentially exposed to raccoon-contaminated environments; and environmental fecal samples collected in raccoon-inhabited areas. This network also supported accurate acquisition of background information and georeferenced data for all collected samples.

### 2.3 Raccoon trapping network

Raccoon distribution was preliminarily assessed using camera traps and community reports from hunters, hikers, and other stakeholders (5). Camera traps were deployed within a 2.5 × 2.5 km grid covering 35 cells, in area with a high probability of raccoon presence (near riverbeds or water bodies) or previously demonstrated to encompass the local raccoon population (5). The camera traps (different models, but all with 1,920 × 1,080p Full HD video resolution, invisible LED, both AA batteries and 6 V external batteries) were set to record 1-min-long videos, without interruption between consecutive videos. Each device was activated for at least a 3-week period to confirm the presence or absence of raccoons, without bait. The videos were then analyzed by trained operators. Confirmed raccoon camera detections were subsequently used to identify priority sites for live trapping.

Live traps (65 × 25 × 25 cm, Tomahawk 205 model) were pre-baited to acclimate animals and set during late afternoon to coincide with nocturnal activity. Baits included fish-flavored cat food, marshmallows, and anchovy paste. Non-target captures were promptly released. Captured raccoons were euthanized according to the state-instituted eradication plan under veterinary supervision, following EU Directive 2010/63/EU Annex IV guidelines. Deep anesthesia was induced with ketamine (10 mg/kg) and medetomidine (0.1 mg/kg) via intramuscular injection. Euthanasia was performed using either firearm or intravenous overdose of Tanax^®^ (0.5 mL/kg), ensuring minimal suffering. Carcasses were submitted within 48 h under refrigerated conditions (+4 °C) to the authors’ institution for necropsy and a thorough health assessment.

### 2.4 Veterinary surveillance

#### 2.4.1 *Baylisascaris procyonis* surveillance and raccoon health assessment

For each raccoon, geographic coordinates of the capture site, sex, age class, and body weight were recorded. In order to evaluate potential relationships among the recorded variables (sex, age, seasonality, and weight) and the presence of *B. procyonis*, statistical analyses were carried out as described in section 2.8. Carcasses underwent gross and histopathological examination to detect and quantify *B. procyonis* infections, assess concurrent pathologies or parasitic infestations, and evaluate intestinal lesions, following previously described protocols (7). All infectious material potentially including *B. procyonis* infective eggs was heat-inactivated at 65 °C for 10 min prior to analysis, for safety reasons (1, 13).

A comprehensive diagnostic assessment was performed on each carcass. Gross pathology included macroscopic examination of the gastrointestinal tract with collection and counting of visible *B. procyonis* adults. The respiratory system was systematically inspected, including the bronchi, thoracic vessels, and lung parenchyma, to detect potential cardio-respiratory parasites. Intestinal tissues were sampled for histopathological evaluation to identify possible penetration of L_3_ larvae into the submucosa and to characterize additional lesions indicative of active infestation.

Standard bacteriological culture methods were applied to individual liver and intestinal samples to test for pathogenic enteric bacteria, including *Salmonella* spp., *Campylobacter* spp., and *Yersinia* spp. (15–17).

Parasitological examinations comprised PCR testing for *Leishmania* spp. on spleen samples; stereomicroscopic analysis of *B. procyonis* adult and immature specimens recovered after sedimentation of rectal fecal material; morphometric identification and quantification of *B. procyonis* eggs in intestinal contents using both qualitative and quantitative flotation methods, including FLOTAC, with differential diagnosis against other ascarid eggs (*Toxocara* spp.). A quali-quantitative copromicroscopic evaluation was performed using multiple techniques (Baermann, FLOTAC, and direct immunofluorescence for *Giardia* spp. and *Cryptosporidium* spp.). Additional stereomicroscopic inspection of coanal washes, urine, or bladder washes was conducted to detect respiratory (e.g., *Eucoleus* spp.) and urinary (e.g., *Pearsonema* spp.) capillariids. Screening for *Trichinella* spp. larvae was carried out on diaphragm and cranial tibial muscle samples through enzymatic digestion in accordance with Regulation (EU) 2015/1375. Molecular diagnostic confirmation of parasitic agents, when required, was achieved using next-generation sequencing (NGS) approaches on adult parasites, larvae, or eggs, following previously described protocols (8).

#### 2.4.2 *Baylisascaris procyonis* surveillance in definitive and potential paratenic hosts other than raccoon

Carcasses of wild animal species susceptible to *B. procyonis* infection and found dead were subjected to investigations aimed at detecting adult parasites in the intestines of potential definitive hosts (e.g., wolves), and larvae in presumed paratenic hosts. All carcasses were submitted with basic background information (species, geographic coordinates, estimated age range) and underwent a standardized diagnostic workup. Necropsy was first performed to document lesions and determine the cause of death. For definitive hosts, intestinal contents were examined for adult ascarids and analyzed qualitatively and quantitatively for *B. procyonis* eggs using the FLOTAC method. Potential paratenic hosts were subjected to pooled-organ sampling (lungs, heart, kidneys, liver, spleen, and brain) for larval detection using the Baermann technique, artificial enzymatic digestion, and brain-squash preparations to detect central nervous system involvement, following Kazacos (1). When macroscopic lesions suggestive of larval migration were observed, histological examination was also performed.

#### 2.4.3 *Baylisascaris procyonis* active surveillance in fecal samples from owned dogs

To extend *B. procyonis* surveillance to potentially exposed domestic animals and species that may be involved in the parasite’s life cycle, fecal samples from privately owned dogs with potential exposure to raccoons, or to raccoon-contaminated environments, were analyzed using qualitative and quantitative coprological methods, including flotation and FLOTAC as previously described (7). All samples were heat-inactivated at 65 °C for 10 min prior to analysis (13). Sampling focused on categories considered at higher risk of environmental exposure, such as hunting dogs, shepherd dogs, and guard dogs. Veterinary practitioners and stakeholders responsible for these dogs were informed and actively involved in sample collection.

#### 2.4.4 Environmental fecal samples collected from raccoon-inhabited areas

Environmental sampling focused on locating raccoon latrines through a structured site-visiting program informed by camera-trapping data and confirmed raccoon sightings. Ten field inspections were conducted by trained operators of the National park and wildlife veterinarians along rivers, streams, and peri-urban areas with confirmed raccoon activity in order to identify potential raccoon latrines or fecal aggregation sites. All peri-urban areas considered at high risk for human exposure, including children’s play-grounds and waste-storage sites, were systematically surveyed. Sites identified during field inspections were subsequently monitored by camera traps to determine the species using them and to differentiate raccoon latrines from those of sympatric mesocarnivores, particularly the European badger (*Meles meles*). Camera trapping was additionally used to investigate hard-to-access peri-urban locations. Latrines identified through these activities were sampled, and fecal material was processed for parasitological examination following the same protocols applied to dog feces.

### 2.5 Spatial GIS analysis for *Baylisascaris procyonis* core zone definition

Spatial analyses were conducted using QGIS software (v3.24.1). The GIS analysis was conceived as an exploratory operational tool to support surveillance and risk-assessment activities rather than as a predictive ecological model. Core areas of raccoon activity were delineated by integrating camera-trap results, live-trap locations, raccoon capture numbers, and the distribution of *B. procyonis*-positive raccoons.

The *B. procyonis* core zone was defined by applying a 5 × 5 km (25 km^2^) buffer around most of the trapping sites, especially around those where parasitologically positive raccoons were captured. Buffer size was selected according to expert opinion and published estimates of raccoon home-range size in different environmental contexts, which may vary substantially depending on habitat characteristics (18). Since the Casentino Valley is characterized by a heterogeneous Mediterranean-mountain landscape including forests, agricultural areas, and peri-urban environments, a conservative buffer approach was adopted in order to support precautionary surveillance and public health activities. Resulting geometries were merged to define the final core zone.

The buffer analysis was subsequently used to identify nearby urban areas, play-grounds, schools, human activities, and livestock facilities potentially relevant for *B. procyonis* exposure. No additional weighting procedure was applied to camera detections, trapping effort, prevalence values, or habitat variables, and uncertainty associated with home-range estimates was not formally modeled.

### 2.6 Genetic characterization of raccoons

Raccoons were genotyped at biparental nuclear (STR, Short Tandem Repeats) and mitochondrial (control region) markers using muscle samples from dead animals. To characterize their genetic makeup and trace single or multiple sources of introduction, our specimens were compared with raccoons collected from captive nuclei and wild populations across Italy and other European countries. The laboratory methods and data analyses are described in detail as previously reported (6).

### 2.7 Public health and stakeholder awareness

Several outreach activities, targeting a wide range of stakeholders, were implemented with the objective of increasing awareness of *B. procyonis* infection and promoting safe handling of carcasses, thereby supporting integrated surveillance and control strategies. Activities used instruments such as web resources, guidelines, educational materials, and multi-sector training events.

### 2.8 Statistical analysis

Statistical analyses were carried out using R software (v 4.5.2) (19). Associations between *B. procyonis* positivity and individual or temporal variables were evaluated using Fisher’s exact test, Pearson’s Chi-squared test, and binary logistic regression. The variables considered were sex, age class, body weight class, and season. Body weight was categorized into three classes (1–3 kg, >3–5 kg, and >5 kg). Odds ratios (ORs), 95% confidence intervals (95% CI), and *p*-values were calculated when applicable. Environmental variables were evaluated descriptively within the GIS-based risk assessment and were not included in the inferential statistical model because sampling and trapping efforts were not designed for predictive spatial analysis.

## 3 Results

### 3.1 Animals and sample collection

One hundred two raccoon carcasses were collected during the study period: 100 animals were culled as part of the eradication program and two were retrieved dead as a result of traumatic events (road-kill and predation respectively). Passive surveillance among paratenic species yielded 169 wild animal carcasses, including 60 mammals and 109 birds. Additionally, 30 wolves (*Canis lupus*) and 15 fecal samples from privately owned dogs (shepherd, hunting, and guard dogs) were included in the analysis as potential alternative definitive hosts.

### 3.2 Raccoon trapping network system

**Figure 1:**
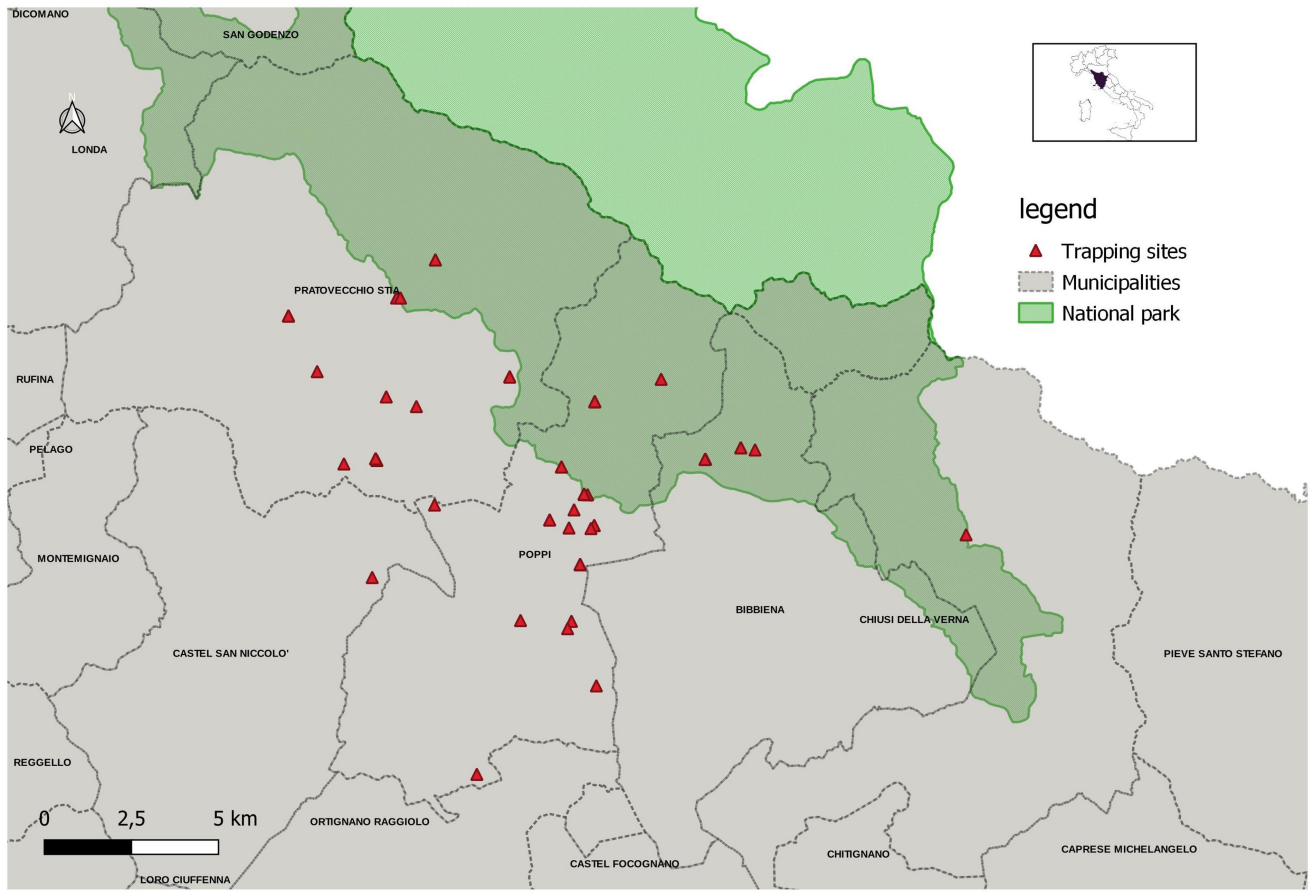
Distribution map of the trapping sites in the area of Casentino Valley. Additional trapping sites located in the municipality of Arezzo are not shown.

The distribution of the 42 trapping sites where the 100 raccoons were trapped and euthanized is shown in Figure 1.

**Figure 2:**
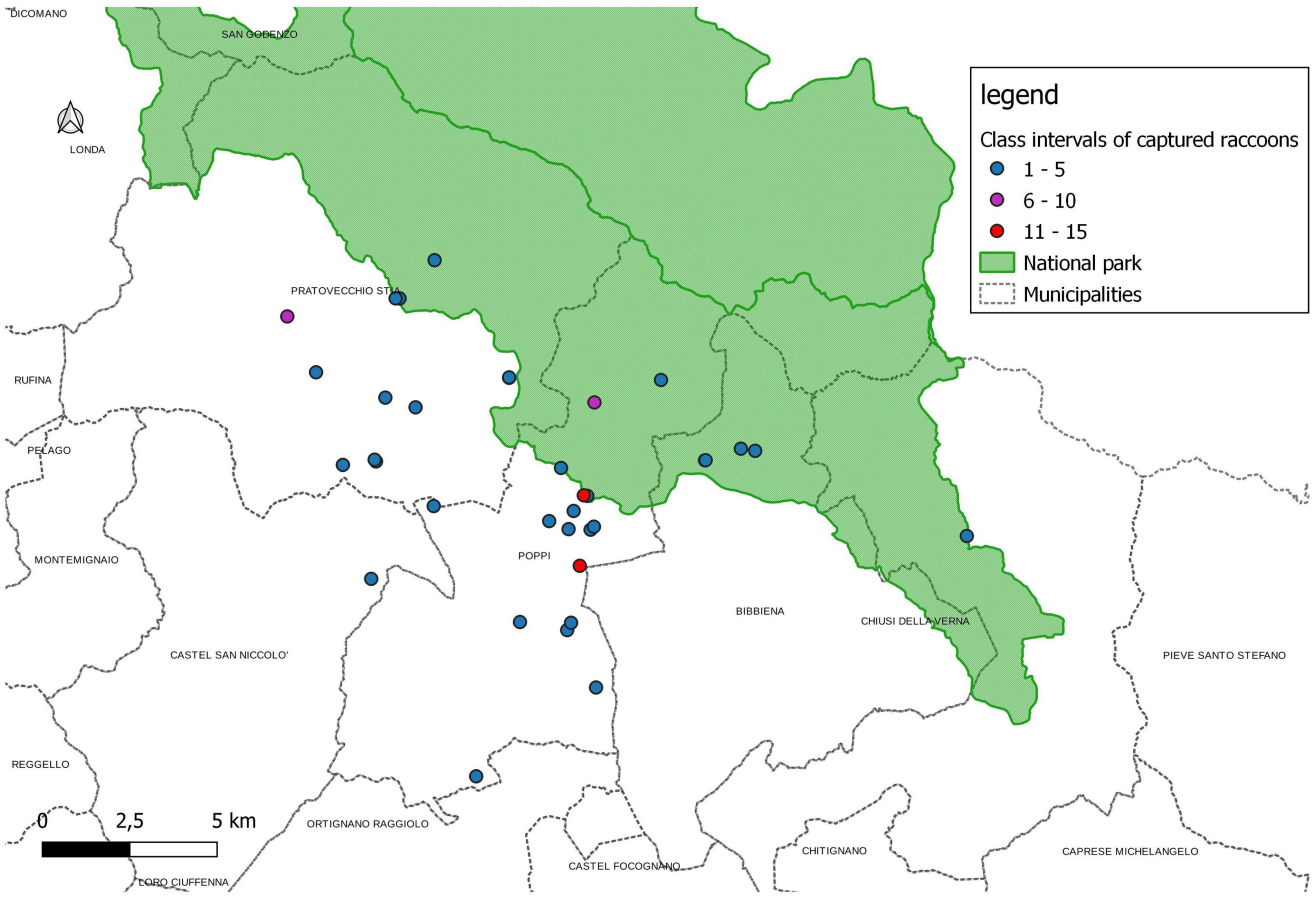
Per-trap class intervals of the number of captured raccoons.

Most traps were positioned in the territories of Pratovecchio Stia and Poppi municipalities where the majority of raccoon sightings occurred. Captured raccoons per trap were grouped into three classes as shown in Figure 2. Between one and five raccoons were captured in more than 90% of trapping sites (38/42), and over half of all raccoons (57/100) were captured in the municipality of Poppi alone.

### 3.3 Veterinary surveillance

#### 3.3.1 *Baylisascaris procyonis* surveillance and raccoon health assessment

All 102 collected raccoon carcasses were examined (50 males, 52 females; 90 adults, 12 juveniles), including euthanized animals and the two recovered carcasses, which showed lesions consistent with predation and mechanical trauma (roadkill), respectively. At parasitological analyses*, B. procyonis* was the most frequently detected parasite (34/102; 33.3%), followed by *Pearsonema* sp. (20/102; 19.6%), other Capillariidae (10/102; 9.8%), Ancylostomatidae (10/102; 9.8%), *Eimeria* sp. (2/102; 1.9%), *Cryptosporidium* sp. (2/102; 1.9%), and *Strongyloides* sp. (1/102; 0.9%). Three raccoons presented coinfections involving *B. procyonis* and either *Eimeria* sp. or Ancylostomatidae. All raccoons tested negative for *Giardia duodenalis*, *Trichinella* spp., and cardiopulmonary nematodes. One individual was positive for *Salmonella enterica* subsp. *diarizonae*. Thirty-eight animals were also tested for *Leishmania* spp., all of which tested negative. Part of these findings was previously reported (7). Qualitative and quantitative results from fecal samples collected from the rectum and urinary sediments are summarized in Table 1.

**Table 1:** Quali-quantitative parasitological results from fecal and urinary sediment samples collected from 102 raccoons.

Parasite	Technique	Prevalence	%	Egg-oocysts/g (m ± sd)*	Egg-oocysts/g (min − max)
*Eimeria* sp.	Flotac	2/102	1.9	4.00 ± 1.16	2–6
*Cryptosporidium* sp.	IF	2/102	1.9	-	-
*Baylisascaris procyonis*	Flotac	32/102**	31.4	3,122.50 ± 7,355.30	2–28,320
Capillariidae	Flotac	10/102	9.8	4.70 ± 17.55	2–96
Ancylostomatidae	Flotac	10/102	9.8	2.50 ± 2.82	2–10
*Strongyloides* sp.	Flotac	1/102	0.9	2.00	0–2
*Pearsonema* sp.	Urine centrifugation/flotation in NaCl	20/102	19.6	–	–

*Confidence rate 95%.

**Two additional animals resulted negative from Flotac, but showed either presence of larvae in the intestinal wall (prepatent period) or a single adult nematode without detectable faecal eggs shedding.

Overall, 34 raccoons were classified as positive for *B. procyonis*. Among these, 32 tested positive by both macroscopic examination and the FLOTAC technique. One animal harbored a single adult worm, resulting in a negative coprological result, whereas another showed evidence of prepatent infection, with larvae detected histologically in the ileal mucosa in the absence of adult parasites. The intensity of *B. procyonis* infection was estimated by counting adult worms in the ileal lumen; results are reported in Table 2. Adult parasites and eggs detected in raccoons were identified through morphometric analysis. Molecular confirmation through NGS/PCR approaches was initially performed on selected isolates during the first detection of the parasite in Italy, as previously described by Lombardo et al. (8), with sequences deposited in the European Nucleotide Archive (ENA, accession number ERZ4009650). Subsequent identifications were based on established morphometric diagnostic criteria. Molecular analyses were thereafter applied only in selected cases involving nematodes or larvae detected in potential paratenic hosts for differential diagnostic purposes.

**Table 2:** Parasite intensity of *Baylisascaris procyonis* (adult nematodes per parasitized animal).

Number of adult parasites (range)	Number of raccoons	%
0–10	8/34*	23.5
10–20	0/34	0.0
20–30	7/34	20.6
30–40	5/34	14.7
40–50	5/34	14.7
50–60	3/34	8.8
60–100	0/34	0.0
>100	6/34**	17.6

*One raccoon showed presence of larvae in the intestinal wall (prepatent period) without harboring adult parasites.

**Three raccoons with parasite intensity higher than 100 parasites showed intestinal obstruction.

**Figure 3:**
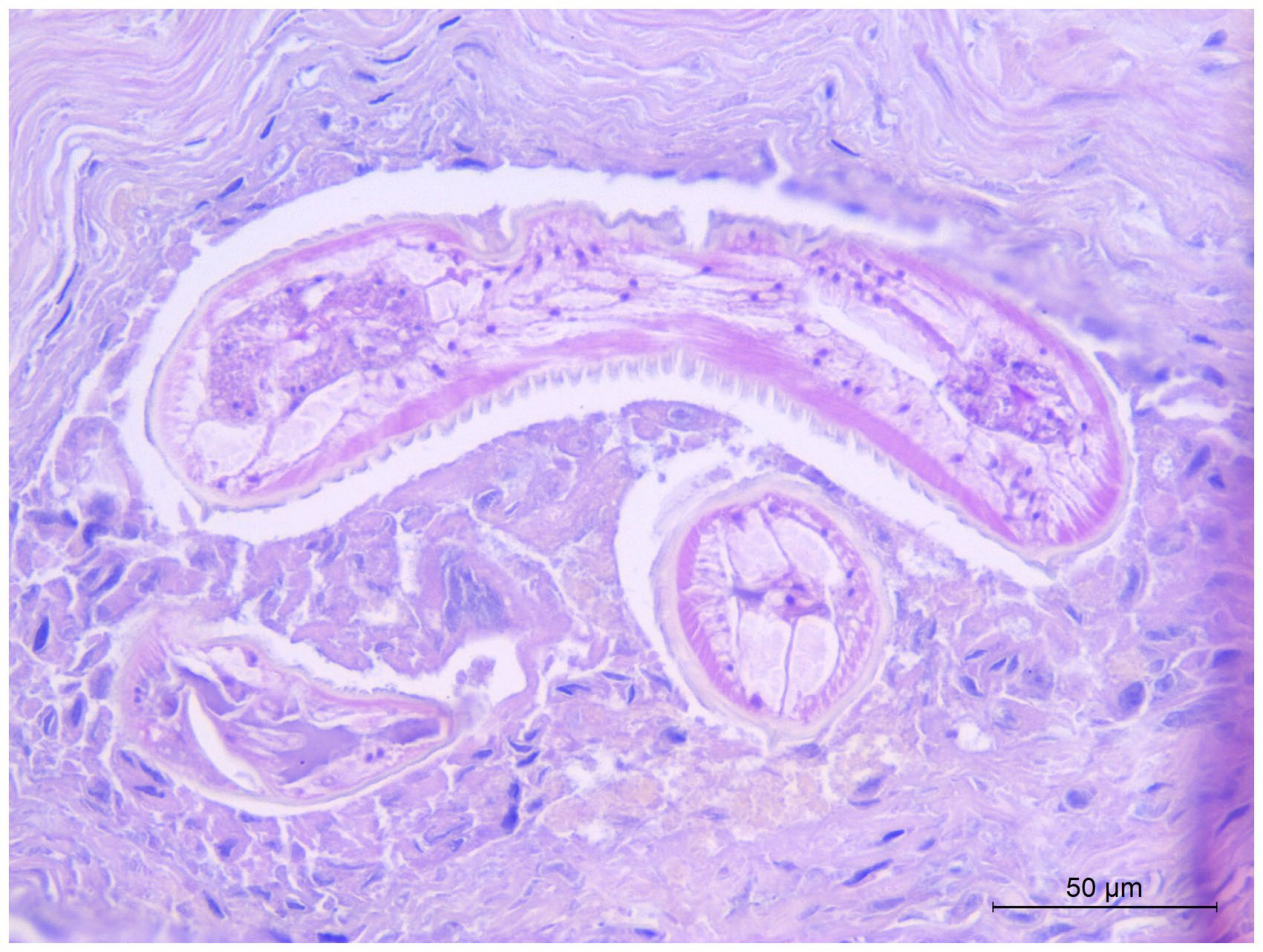
Histological section of raccoon intestine. Larvae of *Baylisascaris procyonis* in the ileal submucosa, H&E, 40×.

In all *B. procyonis-*positive animals, histopathological examination of the ileum revealed eosinophilic enteritis, with microscopic pictures characterized by scattered eosinophils or small eosinophilic aggregates within the crypts, lamina propria, and submucosa (Figure 3). One specimen exhibited a diffuse, full-thickness eosinophilic infiltrate extending into the mesentery. Lymphocytes and plasma cells were observed in three cases. Granulomatous lesions containing larvae embedded in inflammatory exudate (macrophages, lymphocytes, plasma cells, and cellular debris) were identified in the submucosa of three raccoons; two of these animals also harbored adult parasites in the ileal lumen, while one case occurred without detectable adults. No significant intestinal lesions were detected in *B. procyonis*-negative animals.

Histological evaluation of major organs (heart, spleen, liver, lungs, brain, and kidneys) was performed to assess the presence of *larva migrans* stages in raccoons. Findings confirmed the exclusive intestinal localization of the parasite. Occasional extraintestinal lesions (e.g., glomerulonephritis, bronchopneumonia) were observed but were unrelated to parasitic infections.

**Figure 4:**
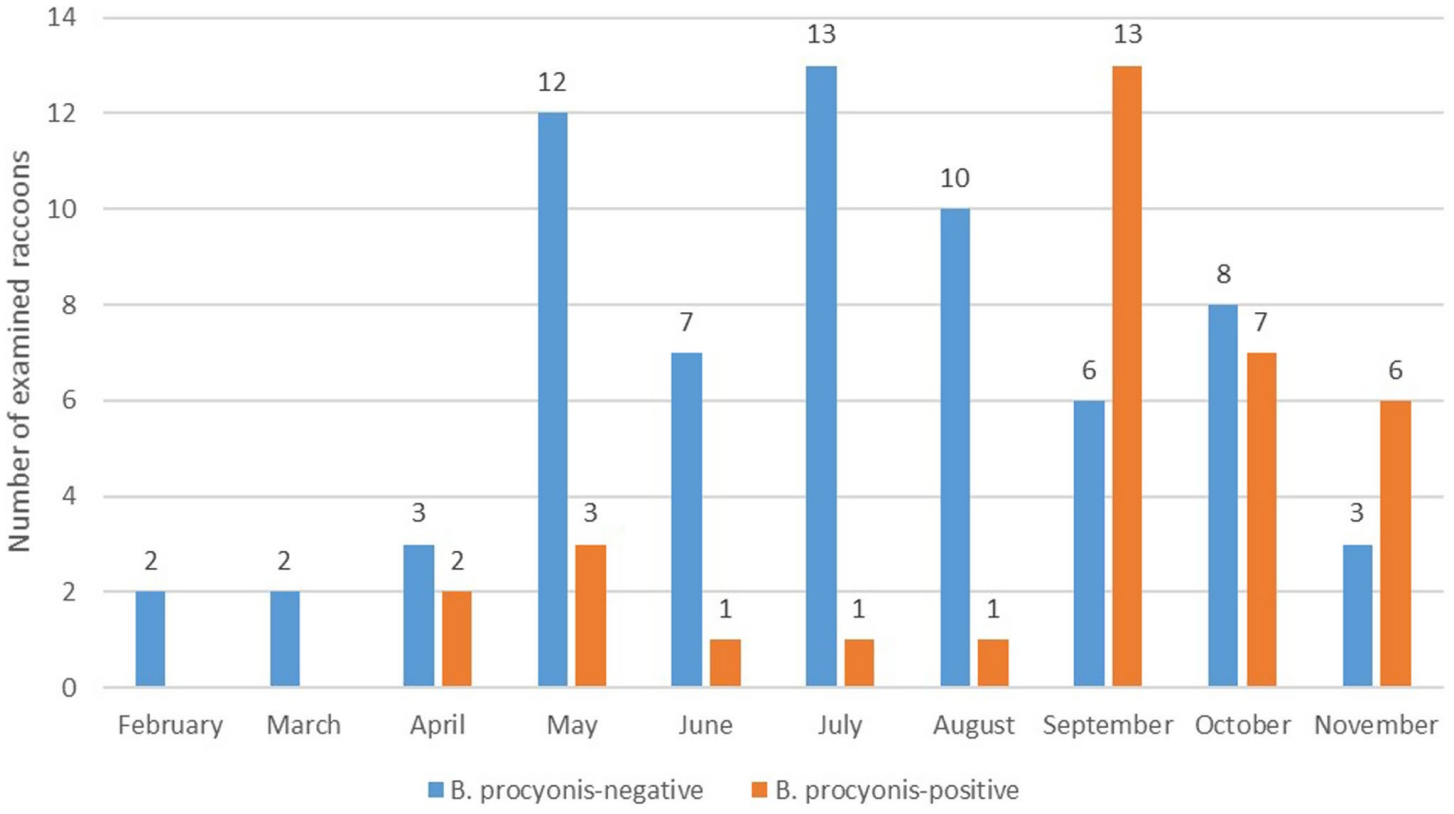
Monthly prevalence of *Baylisascaris procyonis*-positive raccoons.

*B. procyonis* prevalence varied by season, with the highest proportion of positive raccoons detected in autumn and no positive animals detected in winter (Figure 4). Fisher’s exact test showed a significant association between positivity and season (*p* = 0.0446). In the binary logistic regression model, autumn was significantly associated with higher odds of positivity compared with spring and summer (OR = 4.33; 95% CI: 1.34–15.41; *p* = 0.017). No statistically significant association was observed between *B. procyonis* positivity and sex, age class, or body weight class. Juveniles showed a higher, although non-significant, probability of positivity compared with adults (OR = 2.9). Similarly, the lowest body weight class showed the highest proportion of positive animals (8/19; 42.1%), but this association was not statistically significant.

#### 3.3.2 *Baylisascaris procyonis* surveillance in definitive and potential paratenic hosts other than raccoons

A total of 169 carcasses of putative paratenic species were submitted, including 60 mammals—one beech marten (*Martes foina*), two wildcats (*Felis silvestris*), three dormice (*Glis glis*), six crested porcupines (*Hystrix cristata*), 11 badgers (*Meles meles*) and 37 red foxes (*Vulpes vulpes*)—and 109 birds (one common buzzard (*Buteo buteo*), one Eurasian sparrow hawk (*Accipiter nisus*), one Eurasian jay (*Garrulus glandarius*), 11 carrion crows (*Corvus corone*), and 95 Eurasian magpies (*Pica pica*)). In addition, 30 wolf carcasses were examined. Most animals were found dead, with causes of death attributed to traumatic events (e.g., roadkill), suspected poisoning, or infectious disease (e.g., encephalomyocarditis in dormice). Several foxes and magpies were included as part of routine population control and arbovirus surveillance programs, respectively. No evidence of *B. procyonis* infection was detected in the examined animals by parasitological or histological investigations.

Other parasite taxa detected during these examinations included Ancylostomatidae, *Toxocara* sp., *Taenia* sp., *Dipylidium caninum*, *Echinococcus granulosus sensu stricto*, *Angiostrongylus vasorum*, *Eucoleus* sp., *Pearsonema* sp., *Isospora* sp., and *Giardia duodenalis*. Two nematode specimens were subjected to further molecular analyses for precise identification at the species level: an adult nematode from a wolf intestine and a larval stage from badger tissues; the former was identified as *Toxocara canis* and the latter as *Perostrongylus falciformis.*

#### 3.3.3 *Baylisascaris procyonis* active surveillance in feces of owned dogs

A total of 15 fecal samples were submitted and examined using standard flotation techniques and the FLOTAC method. No *B. procyonis* eggs were detected in the examined samples.

#### 3.3.4 Environmental fecal samples collected from raccoon-inhabited areas

Surveys conducted along rivers and streams identified four potential raccoon latrine sites. However, camera trapping, carried out with the same methods used for the raccoons, revealed that all were attributable to badgers. Consistently, fecal samples collected from these sites did not show evidence of *B. procyonis*. Furthermore, no raccoon latrines were identified in peri-urban areas.

### 3.4 Spatial GIS analysis for *Baylisascaris procyonis*-core-zone definition

**Figure 5:**
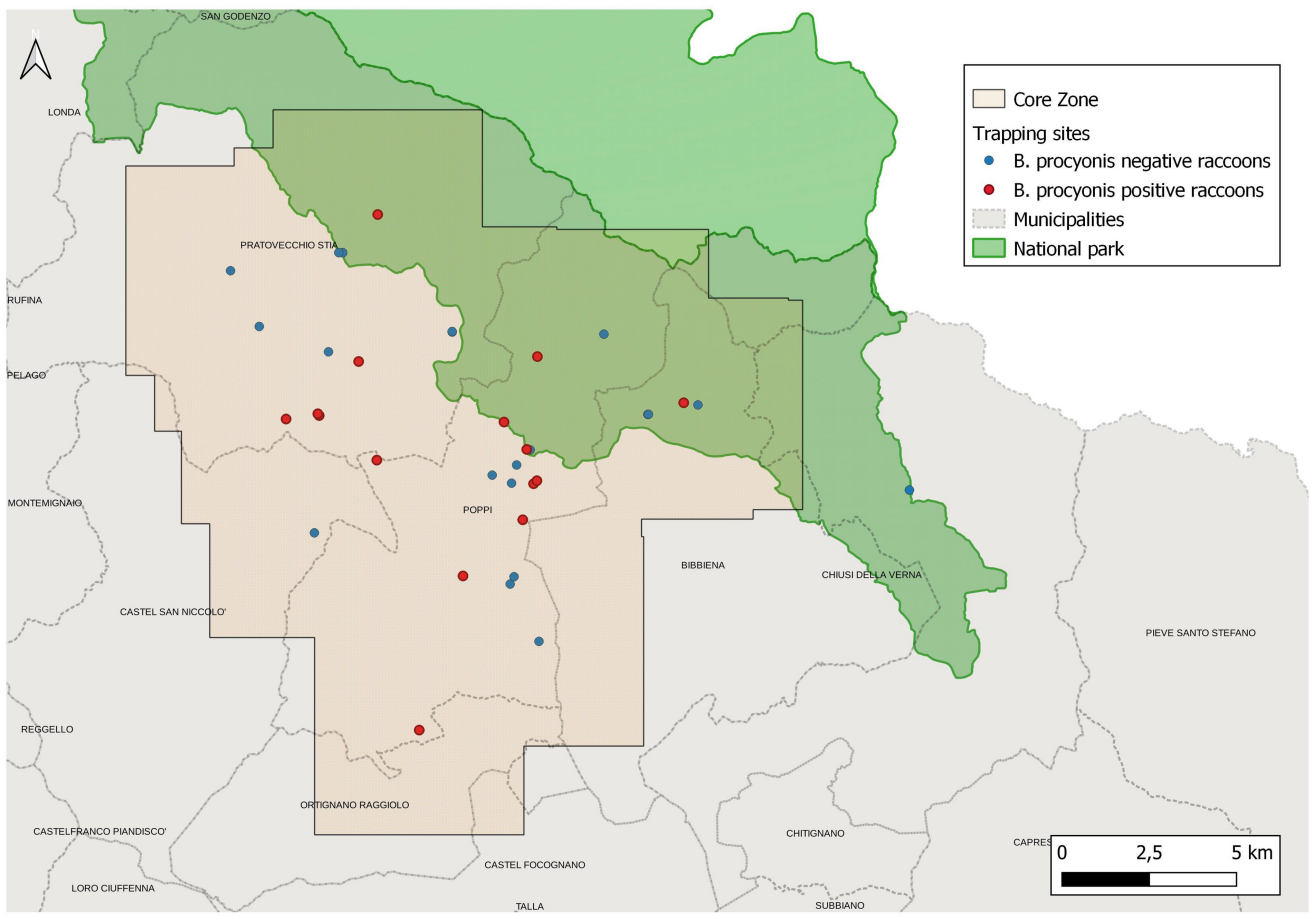
Map of the core zone and the *Baylisascaris procyonis* positive raccoon trapping sites. Red dots indicate the sites where at least one *B. procyonis-*positive raccoon was captured.

The identified core zone, where most of the raccoons were captured, measured about 265 km^2^ and included most of the territories of Poppi and Pratovecchio Stia as well as small parts of the remaining municipalities (Figure 5).

Most of *B. procyonis* positive raccoons (26/34; 76.4%) were captured in the territories of Poppi municipality (Table 3). None of the areas considered at high risk for human exposure (children play-grounds or schools) were adjacent to or within a close range of the *B. procyonis* positive trapping sites.

**Table 3:** Distribution of *Baylisascaris procyonis*-positive traps per municipality.

Municipality	Number of *Baylisascaris procyonis* positive traps/total traps per municipality	% of positive traps
Bibbiena	1/4	25.0%
Castel San Niccolò	0/1	0.0%
Chiusi della Verna	0/1	0.0%
Poppi	9/20	45.0%
Pratovecchio Stia	5/16	31.3%
Total	15/42	35.7%

### 3.5 Genetic characterization of raccoons

The analysis of 11 STR markers and a 550-base-pair long segment in the mitochondrial control region revealed that the raccoons collected in the Casentino Valley derived exclusively from a neighboring private Zoo-Park. The original captive founders had inadvertently escaped in the early 2000s, establishing rapidly a reproductive population in the wild (6). One single mitochondrial haplotype (PLO2a), already found in Europe, was identified in all samples, and no evidence for multiple introductions, either from captive or wild sources, were found.

### 3.6 Public health awareness

The broad outreach campaign implemented to raise awareness of *B. procyonis* infection, promote safe behaviors, and support coordinated surveillance targeted a wide range of stakeholders, including health professionals, veterinarians, hunters, students, local authorities, and the general public, reaching several hundred participants overall. The initiative included the launch of a dedicated Italian web page and the development of regional guidelines to coordinate personnel involved in sample collection. Technical and non-technical informational materials on the safe management of latrines were distributed to hunters’ associations, hospitals, veterinary clinics and diagnostic laboratories. Communication efforts also involved multiple national and international scientific venues, including meetings organized by scientific societies such as Euroraccoon (2023–2024), the Italian Society of Wildlife Ecopathology (SIEF), and the Italian Veterinary Public Health Society (SIVEMP), as well as seminars and a webinar attended by approximately one hundred professionals. Additional targeted training included sessions for hunters, medical staff in high-risk hospital districts, local police forces, university students, and high-school classes. Overall, these integrated activities engaged multiple sectors and ensured wide dissemination of key public health messages related to *B. procyonis* prevention and control.

## 4 Discussion and conclusion

In this study, an integrated surveillance approach for *B. procyonis* was implemented in a recently colonized area of Italy, where a localized population of non-native raccoons established following a single escape event. Across Europe, the expansion of free-ranging invasive raccoon populations has led to growing concern about the emergence of *B. procyonis* as a zoonotic hazard (20). Established populations are well documented in Germany, where the northern-bound expansion of *B. procyonis* infection is closely monitored (21–23). More recently, first detections of *B. procyonis* have been reported in Luxembourg (24), the Netherlands (25), the Czech Republic (26), and isolated areas of France (27), whereas studies from Spain have thus far yielded negative results (28). Despite the increasing number of reports from free-ranging raccoon populations in Europe, information on the occurrence of *B. procyonis* in wildlife species other than raccoons remains scarce. Most European investigations have primarily focused on the definitive host, whereas systematic surveillance of potential paratenic hosts has rarely been implemented. In the present study, potential paratenic hosts were therefore selected based on ecological and taxonomic affinity with wildlife species previously reported as susceptible in North America (1). However, coordinated, integrated surveillance programs targeting invasive species remain rare, and most national efforts still rely on fragmented or opportunistic sampling (29). Within this continental scenario, this work represents the first structured One Health-oriented surveillance initiative targeting *B. procyonis* in Southern Europe, providing a model for early response in newly colonized areas.

The coordinated system integrating wildlife management, diagnostic activities, environmental assessment, and public health outreach provided an integrated framework to support risk assessment and guide mitigation strategies targeting transmission to other animal species and humans. Within a limited geographic area and under defined ecological conditions, such a cross-sectoral approach can be successfully implemented to reduce the risk of zoonotic transmission and to limit the progression of the *B. procyonis* life cycle.

In this context, several complementary actions emerged as key components of an effective response. These included the progressive removal of the raccoon reservoir, systematic assessment of parasite distribution to identify high-risk areas, strengthening of passive surveillance, and prompt diagnostic investigation of paratenic species. Additional measures comprised environmental assessment and safe management of suspected latrines, veterinary monitoring of at-risk dogs as potential bridge hosts, and sustained dissemination activities targeting both professionals and the general public. Together, these elements formed a coordinated One Health framework guiding surveillance and risk mitigation.

Our diagnostic findings are consistent with previous European surveys reporting raccoons as hosts of diverse helminths and protozoa, but identifying *B. procyonis* as the parasite of greatest zoonotic significance (21, 22).

In contrast to previous Italian findings, where no seasonal variation in *B. procyonis* prevalence was observed (7), the present study found a significant association between season and parasite positivity, with higher odds of detection in autumn. This pattern is consistent with seasonal dynamics described in the species’ native range (1), where raccoon activity, juvenile recruitment, and environmental exposure may influence parasite transmission. However, no statistically significant association was observed with sex, age class, or body weight class, although juveniles and lighter animals showed a moderate trend toward higher positivity. Therefore, the observed seasonal pattern should be interpreted cautiously. The limited number of juveniles, the uneven seasonal structure of the sampled population, and the descriptive nature of parasite intensity data prevent firm conclusions on seasonal variation in parasite burden. Overall, these findings suggest a preliminary seasonal signal in *B. procyonis* detection, but further studies with larger and more balanced datasets are needed to clarify its epidemiological significance.

The GIS-based analysis delineated a core area of approximately 265 km^2^, characterized by low human density (average 38 inhabitant/km^2^), and with the population concentrated in a limited number of small towns. Livestock activities involving *B. procyonis* susceptible host species within this area include one large laying-hen farm and several small, backyard holdings of poultry and rabbits. In this regard, no abnormal mortality events or clinical signs attributable to *B. procyonis* infection, such as neurological signs, were observed over the surveillance period.

Importantly, despite extensive passive surveillance across a wide range of potential wild paratenic and definitive hosts, no evidence of *B. procyonis* infection was detected outside raccoons. Neither adult worms nor *larvae migrans* were identified in wild canids, mustelids, felids, rodents, or bird species, and all fecal samples from at-risk owned dogs tested negative. No human cases were recorded, in line with previous observations indicating that autochthonous human baylisascariosis remains extremely rare in Europe despite the expansion of raccoon populations (30, 31). These findings indicate that no evidence of parasite circulation beyond raccoons, nor of onward transmission potentially associated with environmental contamination, was detected during the study period.

During the course of the present investigation, raccoons were no longer present at the local zoo. Therefore, it was not possible to include the captive population in the study. Nevertheless, health control programs for wildlife kept in captivity within zoos or wildlife sanitary shelters should be encouraged as a point of utmost importance for veterinary prevention, especially for alien invasive species.

Environmental surveillance did not identify confirmed raccoon latrines. Several sites initially suspected as such were ultimately attributed to badgers, which can produce latrines morphologically similar to those of raccoons. Previous studies have shown that raccoons living outside North America at low population densities often defecate at dispersed, non-fixed locations rather than at communal latrines (10), making detection in natural settings more difficult. Latrines of raccoons and other mesocarnivores are also recognized as territorial marking and communication sites that may attract conspecific and heterospecific individuals (32, 33). Therefore, when present, latrines may facilitate indirect parasite transmission through repeated environmental exposure to parasitic infective stages. In the present study area, the apparent dispersion of feces and the absence of confirmed raccoon latrines may have reduced localized egg accumulation and, consequently, the intensity of environmental contamination, potentially lowering the risk of accidental ingestion, particularly among children.

The extensive engagement of professional and public audiences was a central component of the surveillance model. Outreach activities reached several hundred individuals, including veterinarians, physicians, wildlife personnel, local police, hunters, and university students and high-school students. These initiatives were essential for harmonizing field activities, promoting appropriate hygiene and environmental management practices, and improving the recognition of *B. procyonis*-related risks. In particular, raising awareness among medical professionals is crucial to ensure that *B. procyonis* is considered among differential diagnoses when compatible clinical presentations arise.

A notable limitation of the present framework is the absence of an EU-based laboratory providing validated serological diagnostics for *B. procyonis*. Since serology represents an important tool for the diagnosis and epidemiological investigation of human baylisascariosis (31), this gap hampers early detection of human exposure and limits the implementation of harmonized surveillance strategies at the European level. Strengthening diagnostic capacity will be essential as raccoon populations are likely to continue expanding across the continent.

The GIS-based delineation of the core zone should also be interpreted cautiously, as it was developed as an operational surveillance tool rather than a formal predictive ecological model. The approach relied on simplified home-range assumptions and did not include weighted habitat suitability analyses or formal uncertainty modelling. Nevertheless, it provided a practical framework for identifying areas requiring intensified surveillance and public health attention during the early phases of invasion management.

Additional limitations of the present study include the opportunistic nature of part of the wildlife sampling, the relatively small number of environmental and domestic animal samples, and the lack of standardized trapping-effort estimates across all investigated areas. These factors may have reduced the probability of detecting low-level circulation outside raccoons. Therefore, negative findings should be interpreted cautiously within the limits of the investigated samples.

Overall, the integrated surveillance model implemented in the Casentino Valley proved effective in defining the distribution of *B. procyonis*, assessing associated risks, and engaging the multiple sectors required to prevent zoonotic transmission. Although current evidence suggests that parasite circulation was mainly confined to raccoons during the study period and has not yet involved other wildlife species, dogs, or humans, continued vigilance is warranted. Recent species distribution and connectivity models predict a significant potential for further south- and east-ward expansion of raccoon populations across Europe, identifying multiple ecological corridors linking Central European populations to Southern Europe (34). This projection underscores that areas currently free of raccoons, including much of Southern Europe, may later become suitable for colonization, thereby increasing the geographic extent of *B. procyonis* risk. In this context, the One Health surveillance approach described here represents a timely and replicable early-warning model for regions susceptible to future colonization events.

Future work should focus on refining surveillance strategies, improving environmental detection methods, and strengthening diagnostic capacity, particularly for human serology. Sustained coordination among wildlife managers, public health authorities, veterinarians, and local communities will be essential to prevent *B. procyonis* from becoming a broader public and animal health concern in Europe.

## Data Availability

The original contributions presented in the study are included in the article/supplementary material, further inquiries can be directed to the corresponding author.
